# Healthcare seeking behavior among patients visiting public primary and secondary healthcare facilities in an urban Indian district: A cross-sectional quantitative analysis

**DOI:** 10.1371/journal.pgph.0001101

**Published:** 2023-09-05

**Authors:** Najiya Fatma, Varun Ramamohan

**Affiliations:** Department of Mechanical Engineering, Indian Institute of Technology Delhi, New Delhi, India; University of Waterloo School of Public Health and Health Systems, CANADA

## Abstract

In this work, we examined healthcare seeking behavior (HSB) of patients visiting public healthcare facilities in an urban context. We conducted a cross-sectional survey across twenty-two primary and secondary public healthcare facilities in the South-west Delhi district in India. The quantitative survey was designed to ascertain from patients at these facilities their HSB—i.e., on what basis patients decide the type of healthcare facility to visit, or which type of medical practitioner to consult. Based on responses from four hundred and forty-nine participants, we observed that factors such as wait time, prior experience with care providers, distance from the facility, and also socioeconomic and demographic factors such as annual income, educational qualification, and gender significantly influenced preferences of patients in choosing healthcare facilities. We used binomial and multinomial logistic regression to determine associations between HSB and socioeconomic and demographic attributes of patients at a 0.05 level of significance. Our statistical analyses revealed that patients in the lower income group preferred to seek treatment from public healthcare facilities (OR = 3.51, 95% CI = (1.65, 7.46)) irrespective of the perceived severity of their illness, while patients in the higher income group favored directly consulting specialized doctors (OR = 2.71, 95% CI = (1.34, 5.51)). Other factors such as having more than two children increased the probability of seeking care from public facilities. This work contributes to the literature by: (a) providing quantitative evidence regarding overall patient HSB, especially at primary and secondary public healthcare facilities, regardless of their presenting illness, (b) eliciting information regarding the pathways followed by patients visiting these facilities while seeking care, and (c) providing operational information regarding the surveyed facilities to facilitate characterizing their utilization. This work can inform policy designed to improve the utilization and quality of care at public primary and secondary healthcare facilities in India.

## 1. Introduction

Healthcare seeking behavior (HSB) involves decisions taken by patients on where to seek care [1, 2]. HSB is influenced by the patient’s illness condition, socioeconomic and demographic characteristics, quality, availability, and accessibility of healthcare services, and pathways chosen to seek care [3]. Healthcare facilities are organized in a hierarchical manner across the globe wherein lower-level facilities provide treatment for common ailments of mild and moderate severity and higher-level facilities focus on specialized care to ensure equitable access to medical services [4]. Despite the hierarchical system, a significant proportion of patients often bypass lower-level facilities and seek care directly at higher-level facilities due to multiple factors [5]. The absence of reliable and accessible points of contact at the recommended facilities, poor quality of care, and lower awareness of the hierarchical system is associated with patients choosing pathways other than those recommended; resulting in disappointment and sometimes impoverishment, especially for those visiting private facilities [6].

The Indian government aims to provide equitable access to quality healthcare, and a key part of this involves establishing primary healthcare facilities as the first point of contact in the event of illness/injury. Despite provision of free medical services at public facilities, curative health services are predominantly (approximately 75%) provided by private, for-profit healthcare providers [7]. Multiple questions thus arise: (a) on what basis do patients decide to seek care from public primary and secondary healthcare facilities in an urban metropolitan district with a significant number of private alternatives, (b) what factors are associated with patients visiting primary and secondary care facilities upon first falling ill instead of specialized public or private facilities such as hospitals, and (c) what pathways do patients adopt to reach a particular healthcare facility. In this study, we attempt to answer these questions by administering an appropriately designed survey to patients actually visiting public primary and secondary care facilities in the South-west district of the urban metropolitan city of New Delhi. Note that answering the above questions comprehensively would involve also surveying patients visiting (a) private facilities providing primary and secondary care, and (b) tertiary care public and/or private facilities upon first falling ill, and (c) potential patients within households (i.e., those not present at the facility). This study thus represents a first step towards answering the above questions.

The determinants of the utilization of public healthcare facilities in the urban Indian context is a complex topic, with several interacting factors: patient HSB, perceptions of quality of care, availability of resources at public facilities, availability of private alternatives, and other socioeconomic and demographic factors. Despite several government initiatives aimed at improving public healthcare services and consequently their utilization, the private sector has been a dominant player in most Indian states [8, 9], with multiple areas even registering five-year declines in the utilization of government services [10]. Several studies found that urban Indian patients preferred private health facilities due to factors such as perceptions of getting better quality of care [11]; however initiatives such as free medications and health financing are improving utilization of public facilities and reducing inequities in access to quality healthcare [12–15]. Utilization of public health facilities, especially in rural Indian areas, was greater among people belonging to lower socioeconomic classes, with socially marginalized communities typically seeking care from informal providers [16]. Further, education level, economic status, and patient standard of living significantly influenced the perception of quality of care and in turn the utilization/non-utilization of public healthcare facilities in rural areas in India [17] as well as in Bangladesh [18]. We provide a detailed summary of the literature in the S1 Table, including a characterization of this study in the last row.

In this study, we collect HSB data from multiple primary and secondary public healthcare facilities in a dense urban metropolitan city with a significant number of private alternatives. Collecting HSB information via in-person visits can yield authentic information from participants actually accessing the system. We also recorded their travel information, which was not reported in similar previous studies. This contributes to the literature on patient pathways as well as on the determinants of healthcare facility utilization. We statistically test the hypothesis that the socioeconomic and demographic attributes of patients impact their HSB. We also report operational information on availability of personnel and other resources and average daily patient loads across healthcare facility networks. Thus, our study provides a quantitatively rigorous analysis of patient HSB and its socioeconomic and demographic determinants in the context of urban public primary and secondary healthcare delivery.

## 2. Methodology

We conducted the cross-sectional survey at twenty-two primary and secondary public healthcare facilities from December 2019 to April 2022 in the South-west Delhi district in India. South-west Delhi is one of the eleven administrative districts of National Capital Territory of Delhi in India with seventy-seven villages in the district [19]. We present South-west Delhi district profile in Table 1. We chose South-west Delhi as the study area owing to two reasons: (a) availability of significantly larger number of primary and secondary public healthcare facilities in comparison to other districts, and (b) proximity to the authors’ institute. We had to suspend the survey twice due to the COVID-19 pandemic and gathered participant responses within a total duration of six months.

**Table 1:** South-west Delhi district profile.

Area (sq. km)	420
Population	2,292,958
Population density (persons per sq. km)	5445
Gender ratio	836
Literacy rate	88.81%

We made in-person visits to three types of healthcare facilities—dispensaries, primary urban health centers (PUHCs), and polyclinics—categorized under primary and secondary level of care under the Delhi public healthcare delivery system [20]. As part of providing the necessary context for understanding the availability of resources at these facilities, which in turn influences patient HSB, their perceptions of the quality of care provided at these facilities, and their utilization, we collected key operational details regarding the services provided at these facilities. These included the different types of services offered by each facility, average patient load per day at different departments, availability of different types of healthcare providers, and other information based on discussion with doctors and data collected from patient records maintained at these facilities. This information is provided in Section 3.1, along with a discussion of the findings.

**Fig 1:**
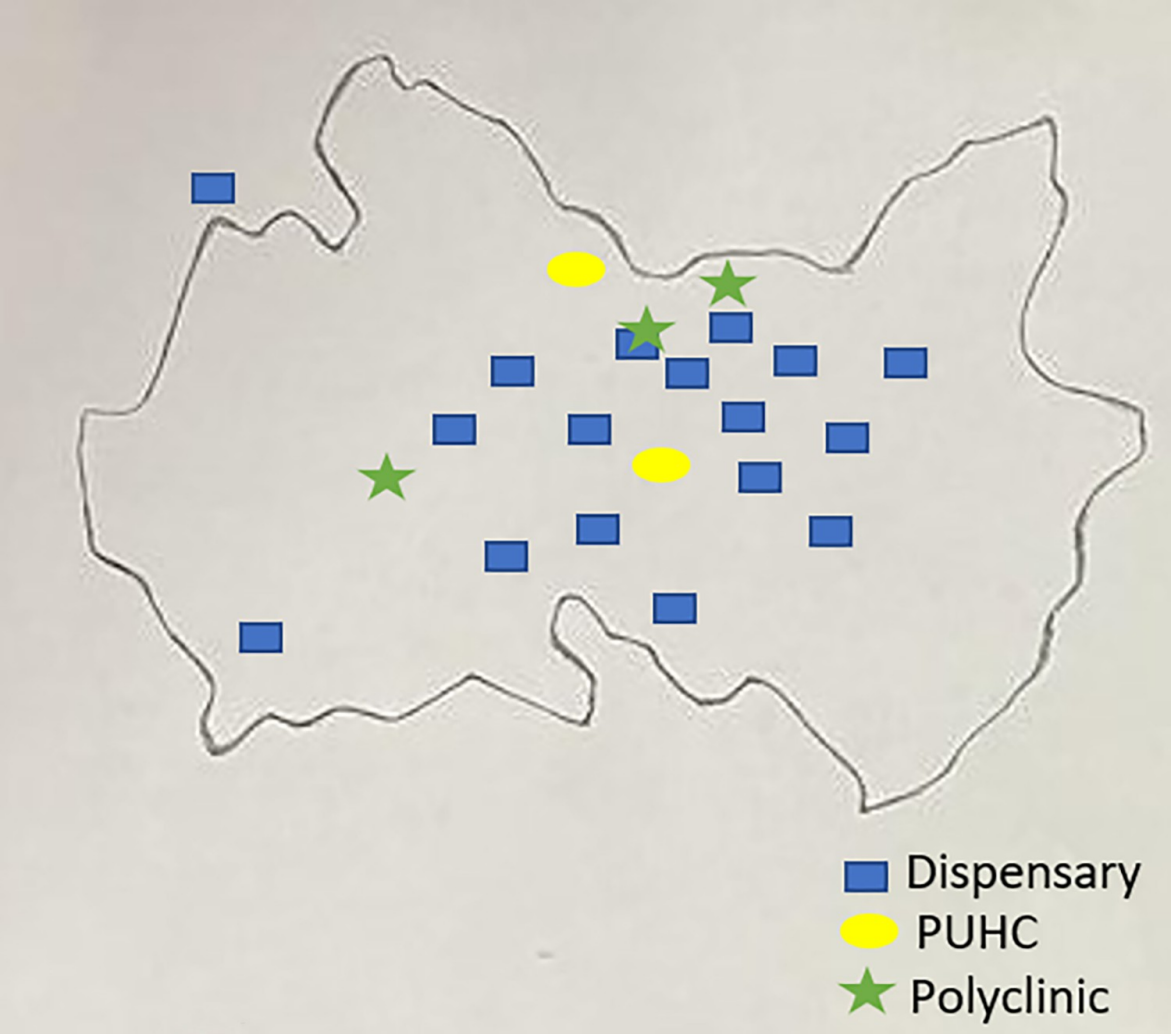
Location of healthcare facilities at the South-west Delhi district.

The Government of NCT of Delhi has a three-tier healthcare delivery system including dispensaries, PUHCs, polyclinics, secondary hospitals, and tertiary care hospitals to provide healthcare services to its target population [20]. Primary healthcare services are available to patients through dispensaries and PUHCs. Polyclinics have been set up for providing specialized services through specialists in medicine, paediatrics, ophthalmology, orthopaedics, gynaecology, ENT, and dermatology. Facilities offering a higher level of care than polyclinics provide a wide range of services across clinical specialties such as surgery, cardiology, nephrology, and urology. We highlight the location of the surveyed public primary and secondary healthcare facilities in Fig 1. Note that a large number of private primary and secondary care clinics as well as tertiary care hospitals are present in South-west Delhi and in Delhi NCR in general; however, the exact number is not in our knowledge publicly available.

### 2.1 Survey data collection

We conducted in-person face-to-face interviews using a close-ended survey questionnaire from respondents present at healthcare facilities. The survey questionnaire itself and a description of the rationale behind each question is provided in the S1 Text, with the survey questionnaire depicted in S1 Fig. We used the random sampling technique to select the dispensaries, PUHCs, and polyclinics in the Southwest Delhi district in India. We visited 17 dispensaries (out of 23), two PUHCs (out of 7), and three polyclinics (out of 4) to collect survey responses. The dispensaries, PUHCs, and the polyclinics function independently in the healthcare network; however, patients from the dispensaries and the PUHCs are occasionally referred to the polyclinics if they need specialist consultations. We asked respondents to choose from the available options in the questionnaire by ticking or encircling their desired category. Participation was voluntary, and respondents indicated their willingness to participate in a consent form attached to each questionnaire. We observed an overall response rate of ninety percent. We read out questions to patients during the interview and ensured that participation of respondents did not affect regular healthcare delivery operations. Initially we conducted a pilot survey among forty respondents and based on participant responses, we modified and prepared the final version of the questionnaire. We did not collect any personally identifiable information from patients.

### 2.2 Survey administration methodology

We considered patients eligible to participate in the survey if they were: (a) aged greater than 18 years, (b) not severely ill and therefore not in a position to respond, and (c) idle and waiting for their consultation in the queue. We assigned numerical codes to each response category included in the survey questionnaire and compiled participant responses in a single Microsoft Excel sheet. In case of discrepancies or missing responses, we discarded the entire response of the particular respondent.

The Institutional Review Board at the Indian Institute of Technology New Delhi approved the study protocol with approval number IITD-IEC-ID-P064 on November 25, 2019. We asked each potential respondent to sign on the participant informed consent form (PICF) prior to the data collection process. We estimated the final sample size for the patient survey to be four hundred and forty-nine using a 95% confidence interval, margin of error of 0.05, a response rate of 95%, and an eligibility rate of 90%. We provide the statistical details regarding the sample size estimation procedure in the S2 Text. We randomly selected patients present at the facility from among those who satisfied the inclusion criteria. Different sampling techniques such as stratified random sampling [21], systematic random sampling [22], and response driven sampling [23] were previously used for selecting study respondents.

### 2.3 HSB modelling

**Fig 2:**
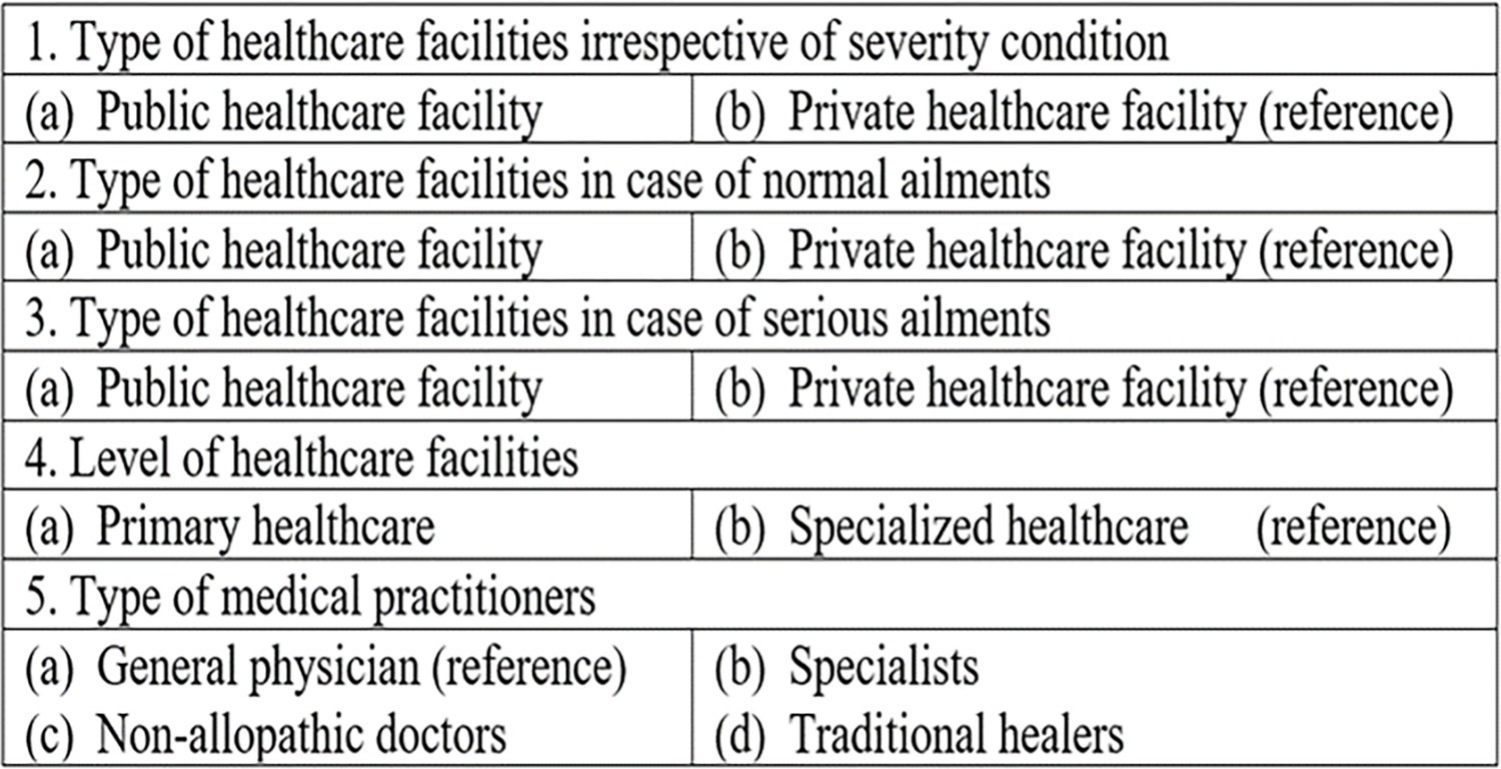
List of dependent variables for logistic regression with reference categories.

We analyzed the relationship between patient HSB and their socioeconomic and demographic attributes using two logistic regression techniques: (a) binomial and (b) multinomial, depending upon number of categories of response variables. We provide the list of dependent variables for analyzing HSB of patients along with associated reference categories in Fig 2. We did not perform logistic regression for the data collected for question A.1 provided in S1 Fig because we encouraged patients to choose more than one option in their response for this particular question.

We developed five logistic regression models: four binomial (1–4 in Fig 2) and one multinomial (5 in additional file—Fig 2) for analyzing HSB of patients. Independent variables were patient attributes including gender, age, marital status, education level, annual income level, occupational status, and number of children. The logistic regression analyses were performed on the R statistical computing platform [24]. All analyses were carried out with a level of significance of 0.05. Prior to conducting the logistic regression analyses, we estimated the correlation matrix for the independent variables to assess the presence of multicollinearity. Based on the correlation matrix, we observed that marital status was highly correlated with age, employment status, and number of children, with correlation estimates of 0.48, 0.58, and 0.77, respectively. While the reported correlation estimates are significant, they are below the generally agreed rule of thumb criterion used in Midi et al. [25] and Senaviratna & Cooray [26] that outline that multicollinearity may be considered to be significant if the correlation coefficient for two variables exceeds 0.8. The authors proposed addressing such multicollinearities by omitting one of the correlated variables from the regression model and then checking how the Akaike’s information criteria (AIC) scores change. Going by this approach, we observed relatively small differences in AIC scores and the estimates of the statistically significant parameters.

## 3. Results

### 3.1 Descriptive analysis

We begin by summarizing the observations regarding operations at the surveyed facilities. In Table 2 below, we provide operational characteristics from two representative dispensaries, PUHCs, and polyclinics at South-west Delhi.

**Table 2:** Operational configurations of primary and secondary public healthcare facilities.

Healthcare facility type		Average daily OPD load, general medicine	Specialist services: number of specialists (daily average patient load)							ANC services	Immunization services	Average number of general physicians, lab technician, receptionist, pharmacist, ANC nurse
			Gynaecology	Ophthalmology	Orthopaedics	Paediatrics	Dermatology	ENT	Surgery			
**Dispensaries (23)**	**1**	190	NA							40–50	45–55	2, 1, 1, 1, 2
	**2**	85	NA							15–40	20–40	1, 1, 1, 1, 1
**PUHCs (7)**	**1**	130	NA							50–60	60–70	2,1,1,1, 1, 2
	**2**	100	1 (60–70)	NA						40–50	50–60	1,1,1,1,1, 1
**Polyclinics (4)**	**1**	210	1 (60–70)	1 (60–80)	1 (100–110)	1 (70–80)	1(120–130)	1 (80–85)	1 (40–45)	50–60	60–70	2,1,1,1, 1, 2
	**2**	160	1 (20–30)	1 (70–90)	1 (120–150)	1 (80–100)	1 (75–80)	1 (80–90)	1 (30–40)	50–60	60–70	1,1,1,1, 1, 2

Information collected from in-person visits and consultations with doctors at the facilities.

OPD = outpatient department; ENT: ear, nose and throat; ANC = antenatal care; PUHC = primary urban healthcare centers.

In general, we observed that there is significant variation in patient loads across the surveyed facilities. Further, the low consultation times with doctors (i.e., average consultation durations of less than a minute) implies that the observed patient loads may be considered to be low, leading to underutilization (from an operational perspective) of doctors, in turn meaning that the proportion of time spent by these providers actually providing medical care is less than 40% on average. These findings are corroborated by operational studies conducted for PHCs [27] and CHCs [28] in semi-urban and rural areas, who also observe low operational utilization of medical staff in these facilities.

We estimated the final sample size for the patient survey to be four hundred and forty-nine using 95% confidence interval, margin error of 0.05, a response rate of 95%, and an eligibility rate of 90%. In order to depict how the surveyed population compares with that of Delhi, we also provide the proportion of patients in each category in Table 3 for a given patient characteristic in the Delhi region as a whole, where available. In our surveyed sample, the majority of patients were female (70.67%) and married (77.56%). In terms of education, approximately 18.88% of patients did not receive any form of formal education and among patients with formal education, almost a third were undergraduates. The highest proportion of patients visiting public healthcare facilities were homemakers (38.44%) followed by professionally employed persons (36.67%). A significant proportion of patients (24.22%) preferred not to reveal their income level.

**Table 3:** Participant profile characteristics at South-west Delhi public healthcare facilities.

Patient profile*		Percentage (out of 100)	Delhi
Gender	Male	29.33 (132)	53.53
	Female	70.67 (317)	46.46
Age	Between 18–29	36.67 (164)	32.56
	Between 30–39	29.55 (132)	26.13
	Between 40–49	14.67 (66)	19.06
	Between 50–59	11.78 (54)	11.35
	60+	7.33 (33)	10.87
Marital status	Married	77.56 (348)	48.61
	Unmarried	22.44 (101)	51.38
Highest level of education	No formal education	18.88 (85)	NA
	Upto 10^th^ grade	16.22 (73)	86.62
	Upto 12^th^ grade	17.77 (80)	6.59
	Undergraduate degree	33.11 (148)	5.94
	Post-graduate degree	14.02 (63)	0.83
Annual Income	Prefer not to say	24.22 (109)	NA
	Upto USD 11,500	26.67 (120)	42.5
	USD 11,500 –USD 23,000	22.89 (103)	20.5
	USD 23,000 –USD 46,000	14.67 (66)	14
	Above USD 46,000	11.55 (51)	23
Occupational status	Student	15.56 (70)	NA
	Homemaker	38.44 (172)	NA
	Employed	36.67 (165)	NA
	Unemployed	9.33 (42)	NA
Number of children	0	30.44 (137)	NA
	1	22.01 (99)	NA
	2	29.11 (131)	NA
	3+	18.44 (82)	NA

*Generated from a field survey of 449 respondents. Education up to 10^th^ grade and 12^th^ grade are significant milestones in the Indian school education system and are formally recognized by all education boards. We converted annual income in INR to US dollars after adjusting for purchasing power parity. In occupational status, students consisted of all those who completed education level as described in categories included in highest level of education. NA: not available.

**Fig 3:**
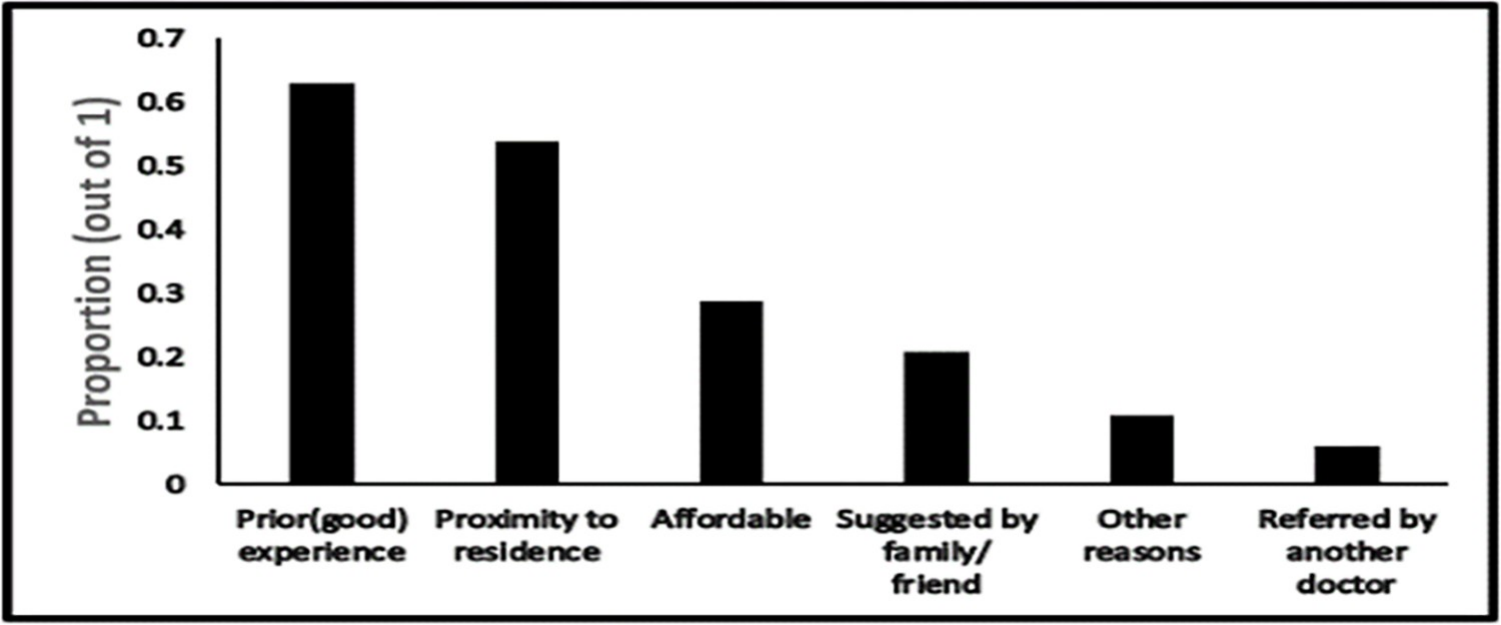
Reasons for visiting public healthcare facilities.

Good prior experiences in terms of provider attitudes and trust in the quality of medical services motivated a significant proportion of patients (62.22%) to visit the surveyed primary or secondary public healthcare facilities (question A.1 in S1 Fig). Other reasons including cleanliness, lesser wait times at healthcare facilities, and provision of free laboratory services prompted patients to return to the same facility. Proximity to healthcare facilities from the patient’s residence also increased the likelihood of seeking care from a given health facility (Fig 3).

**Fig 4:**
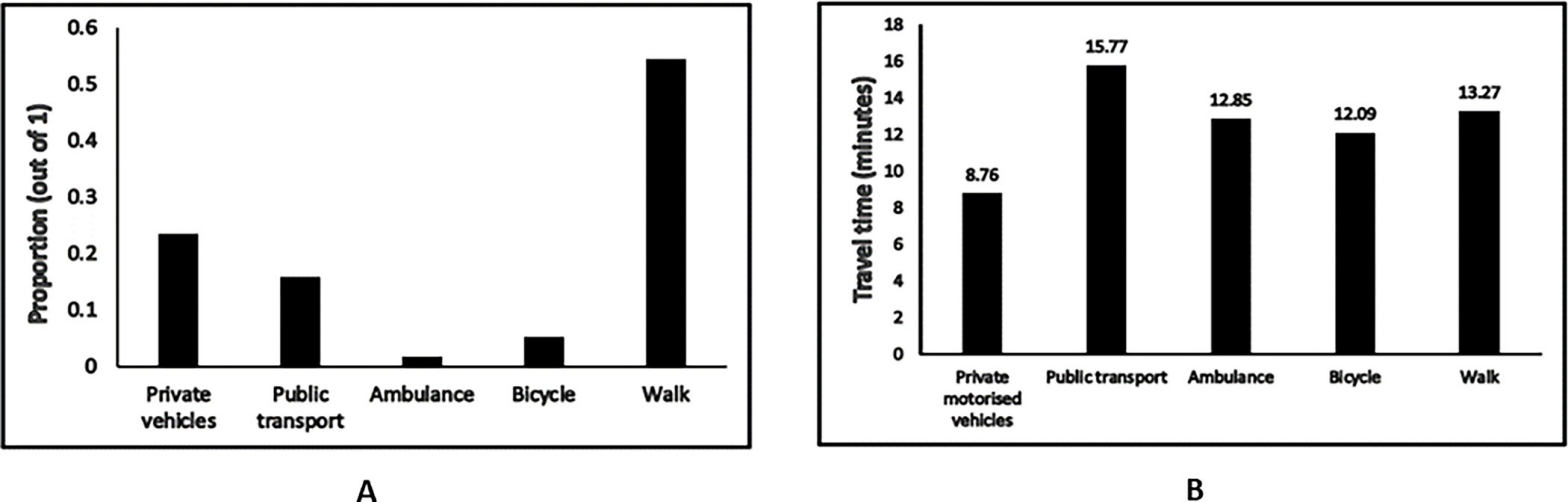
A: Different modes of transportations. B. Average travel time to reach healthcare facilities.

The highest proportion of patients (54.22%) walked to the health facility, which may indicate reasonably quick accessibility to primary and secondary care within the existing healthcare delivery system in the region studied. We observed from Fig 4A that a small proportion of patients (approximately 1.55%) were carried on ambulances to healthcare facilities. These ambulance services were arranged by the patients themselves and were not provided by these facilities as it is not part of their service mandate.

The reported travel time to reach health facilty was lowest for patients visiting healthcare facilities in private motorized vehicles (mean = 8.76 minutes, standard deviation [SD] = 4.75 minutes), followed by patients with bicycles (mean = 12.09 minutes, SD = 5.24 minutes). We summarize the average travel time statistics in Fig 4B, where we observe patients visting healthcare facilities via public transport took the longest time (mean = 15.77 minutes, SD = 10.17 minutes) to reach the health facility.

**Fig 5:**
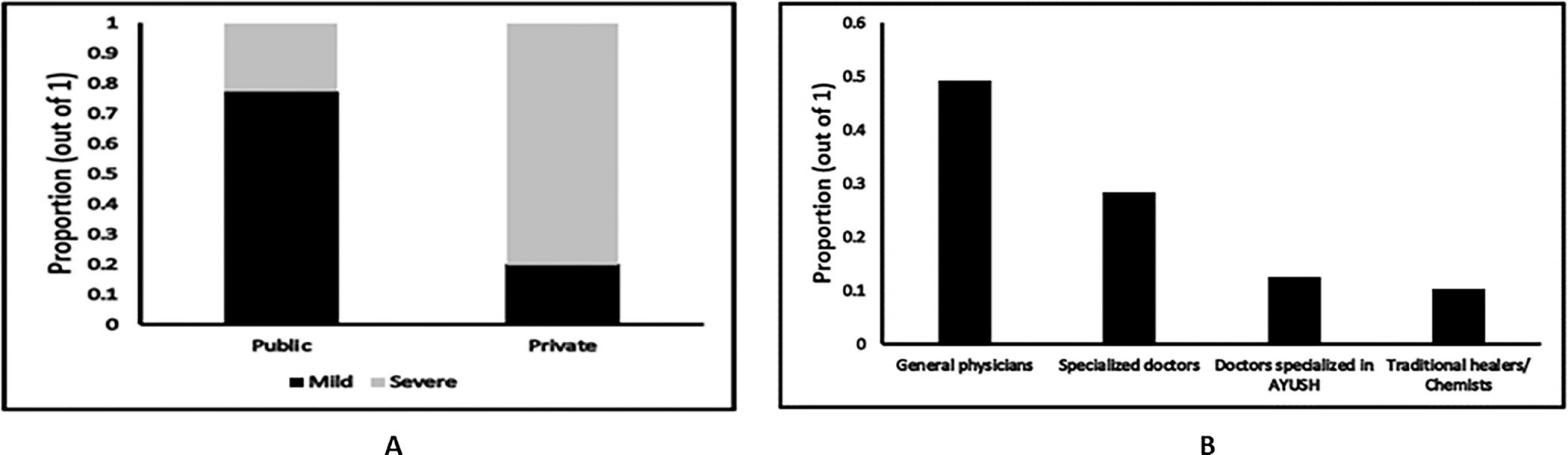
A. Preferences for different types of healthcare facilities for a first visit. B. Patient preferences for different types of providers for the first visit. AYUSH: Ayurveda, Yoga and Naturopathy, Unani, Siddha, and Homeopathy.

Next, we see that a significant proportion of patients (49.11%) favored visiting public healthcare facilities for any health-related issue regardless of their perceived severity of the disease condition. Approximately 30.22% chose the healthcare facility on the basis of the perceived severity of their illness. For illnesses of perceived mild severity, a significantly larger proportion of patients (77.20%) frequently visited public healthcare facilities. For illnesses of perceived high severity and for chronic illness, 80.14% of the patients surveyed sought care from private healthcare facilities. Upon further enquiry, we found that shorter wait times before admission, especially at specialized levels of care, was the primary factor that influenced patients in their choice of private facilities, even though medical services were provided at significantly higher costs in these facilities. We report the inclination of patients towards different types of healthcare facilities in cases of illnesses of perceived mild and high severity in Fig 5A.

Next, regardless of the perceived severity of their illness, we observed that a substantial proportion of patients (40.44%) preferred to make a first visit to specialized healthcare facilities including clinics and hospitals. We also examined patient preferences for consulting different types of healthcare providers. Most patients (49.11%) chose to consult general physicians followed by specialized doctors (28.22%) upon first feeling unwell (Fig 5B).

Interestingly, 10.22% of patients also sought care from traditional healers and among these respondents, majority (2.5 times higher) were female patients without any formal education (41.73%). Such healthcare practices indicate limited health literacy and lower trust in formal care among these categories of patients. A small proportion of patients reported that they consulted traditional healers after not finding relief from treatments suggested by formally trained healthcare providers.

### 3.2 HSB: Inferential analysis

Before conducting the BLR and MLR analyses, we formulated the research question and corresponding research hypotheses associated with each model. We provide the research question associated with the null and the alternate hypothesis and the final BLR and MLR model with the significant variables associated with each model in the S3 Text. We report results from the most parsimonious models along with the percentage change in AIC scores in Tables 4 and 5 for the BLR and MLR analyses, respectively.

**Table 4:** Results from BLR modelling of HSB as a function of socioeconomic and demographic variables.

Research Question	Significant variables	Significant Categories	β (SE)	*z*-value	*P*(>|*z*|)	*OR* (*LL, UL*)
*R1*: Type of healthcare facility: public/private (reference = private)	Gender	Gender (2)	-0.79 (0.35)	-2.24	0.02	0.45 (0.25, 0.80)
	Reference = Male					
	Education level	Education level (2)	0.95 (0.59)	1.60	0.05	2.60 (0.97, 6.97)
	Reference = No formal education					
	Annual income	Annual income (2)	1.25 (0.45)	2.74	0.00	3.51 (1.65, 7.46)
	Reference = Upto USD 11,500	Annual income (5)	-1.43 (0.57)	-2.50	0.01	0.23 (0.09, 0.60)
	Number of children	Number of children (3)	1.39 (0.55)	2.49	0.01	4.05 (1.61, 10.16)
	Reference = 0	Number of children (4)	1.22 (0.56)	1.90	0.05	3.40 (1.34, 8.60)
	Employment status	Employment status (2)	1.15 (0.56)	2.03	0.04	3.16 (1.24, 8.03)
	Reference = Student					
	Δ AIC score (removed marital status)	0.10%				
*R2*: Type of healthcare facility with illness conditions of perceived mild severity: public/private) (reference = private)		Intercept	1.26 (0.48)	2.63	0.00	3.47 (1.88, 6.41)
	Gender	Gender (2)	-0.69 (0.27)	-2.48	0.01	0.58 (0.38, 0.89)
	Reference = male					
	Education level	Education level (2)	0.76 (0.44)	1.71	0.04	2.35 (1.16, 4.76)
	Reference = No formal education					
	Annual income	Annual income (4)	-0.83 (0.40)	-2.06	0.03	0.43 (0.23, 0.79)
	Reference = Upto USD 11,500	Annual income (5)	-0.95 (0.41)	-2.32	0.01	0.38 (0.20, 0.72)
	Δ AIC score (removed marital status)	0.31%				
*R3*: Type of healthcare facility with illness conditions of perceived high severity: public/private (reference = private)	Annual income	Annual income (2)	0.72 (0.32)	2.23	0.02	2.08 (1.22, 3.55)
	Reference = Upto USD 11,500	Annual income (5)	-1.09 (0.42)	-2.56	0.01	0.33 (0.16, 0.68)
	Employment status	Employment status (3)	-0.62 (0.38)	-1.63	0.01	0.50 (0.27, 0.90)
	Reference = Student					
	Number of children	Number of children (3)	0.92 (0.35)	2.61	0.00	3.16 (1.57, 6.37)
	Reference = 0	Number of children (4)	0.89 (0.42)	2.12	0.03	3.05 (1.38, 6.72)
	Δ AIC score (removed marital status)	0.18%				
*R4*: Type of healthcare facility: primary care or specialized (reference = specialized)	Education level	Education level (4)	-1.05 (0.37)	-2.81	0.00	0.34 (0.18, 0.64)
	Reference = No formal education	Education level (5)	-0.98 (0.45)	-2.18	0.02	0.37 (0.17, 0.78)
	Annual income	Annual income (2)	0.76 (0.31)	2.39	0.01	2.14 (1.27, 3.63)
	Reference = Upto USD 11,500					
	Employment status	Employment status (3)	-0.78 (0.37)	-2.09	0.03	0.45 (0.24, 0.84)
	Reference = Student					
	Number of children	Number of children (3)	0.94 (0.39)	2.38	0.01	2.57 (1.34, 4.94)
	Reference = 0	Number of children (4)	0.81 (0.37)	2.21	0.02	2.26 (1.23, 4.16)
	Δ AIC score (removed marital status)	0.21%				

*β: Estimated regression coefficients; SE:* Standard error; *OR*: Odds ratio; *Lower limit (LL) and upper limit (UL) at* 95% *CI*; Δ AIC: Percentage change in AIC

**Table 5:** Results from MLR modelling of HSB as a function of socioeconomic and demographic variables.

MLR model	Attributes	Significant variables	Significant categories	β (SE)	z-value	*P*(>|*z*|)	*OR* (*LL, UL*)
Type of doctor Ref = General physician	Specialists	Annual income	Annual income (4)	1.00 (0.43)	2.30	0.02	2.71 (1.34, 5.51)
		Reference = Up to USD 11,500					
		Number of children	Number of children (3)	-1.17 (0.40)	-2.89	0.00	0.31 (0.17, 0.59)
		Reference = 0	Number of children (4)	-0.94 (0.49)	-1.91	0.05	0.39 (0.17, 0.87)
	Doctors formally trained in traditional medicine		Intercept	-3.10 (0.80)	-3.85	0.00	0.04 (0.01, 0.17)
		Employment status	Employment status (4)	2.59 (0.74)	3.47	0.00	13.32(3.96, 45.02)
		Reference = Student					
		Age	Age (2)	1.15 (0.52)	2.21	0.02	3.15 (1.34, 7.42)
		Reference = 18–29	Age (3)	1.79 (0.62)	2.86	0.00	5.98 (2.16, 16.60)
			Age (4)	1.84 (0.62)	2.92	0.00	6.29 (2.27, 17.45)
	Informal traditional healers	Employment status	Employment status (4)	1.17 (0.78)	1.49	0.05	3.22 (0.89, 11.62)
		Reference = Student					
		Age	Age (4)	1.46 (0.63)	2.92	0.02	4.30 (1.53, 12.13)
		Reference = 18–29					
		Education level	Education level (4)	-1.54 (0.59)	-2.59	0.00	0.21 (0.08, 0.56)
		Reference = No formal education	Education level (5)	-1.44 (0.79)	-1.80	0.05	0.23 (0.06, 0.86)
		Δ AIC score (removed marital status)	0.21%				

From Table 4, we observed that patients with higher annual income were more likely to visit private healthcare facilities in comparison to public healthcare facilities irrespective of the perceived severity of their illness condition. Relatively speaking, patients with annual incomes above USD 46,000 were 4.34 times more likely to visit private healthcare facilities in comparison to patients with annual income up to USD 11,500. Further, we also observed that patients with higher education levels were less likely to visit a public healthcare facility (0.38 times less likely) in comparison to patients with no formal education. This was corroborated by patient comments during the survey, where they indicated that they also preferred to visit a private healthcare facility because of shorter waiting times before receiving care. We also observed that public healthcare facilities were 3.40 times more favored by patients having three or more than three children in comparison to patients with no children.

We see from Table 4 that patient gender had a significant association with patient HSB, with female patients preferring to visit a private healthcare facility 1.72 times more than a public facility in case of illness conditions of perceived mild severity. This observation was also conveyed to us by a few male patients, who indicated that their spouses preferred visiting private healthcare facilities even in cases of mild ailments. Other patient attributes significantly affecting HSB for illness conditions of perceived mild severity were education and annual income.

Employment status, annual level of income, and number of children were notable factors influencing patient HSB for illness conditions of perceived high severity. We also see that employed patients with higher levels of income were more likely to visit a private healthcare facility for getting treatment for their medical condition. The odds of visiting a private healthcare facility increased by 2 times and 3.03 times among patients with a source of employment and having an annual income greater than USD 46,000 in comparison to students and patients without any source of income, respectively. Patients having two or more than two children were 3.16 times and 3.05 times more likely to visit public healthcare facilities in comparison to patients without children.

We report parameter estimates of significant variables for *R4* in Table 4. Higher education levels along with higher annual incomes were associated with increased odds of visiting specialized healthcare facilities such as clinics and hospitals. Patients with undergraduate and post-graduate degrees were 2.94 times and 2.70 times more likely to visit specialized facilities directly in comparison to patients without any formal education.

We formulated one MLR based hypothesis to examine the association between the socioeconomic and demographic factors associated with patient preferences for seeking care–upon first falling ill—from different types of medical practitioners such as general physicians, specialized doctors, doctors formally trained in traditional medicine, and informal traditional healers. As there were more than two categories for the response variable, we implemented the MLR modelling technique. We formulated *R5* in a manner similar to *R1*. We provide the statistical equations for the MLR based analysis in the S3 Text. We report the parameters of significant independent variables in Table 5.

We observed that patients with higher annual income levels preferred directly consulting specialized practitioners. Specifically, patients with incomes between USD 23,000–46,000 were 2.71 times more likely to visit specialized doctors in comparison with patients with annual income level up to USD 11,500. Further, patients with two or more children were 3.22 times and 2.56 times more likely to visit a general physician in comparison to patients without any children. Unemployed patients were 3.22 times more likely to visit traditional healers in comparison to students.

Responses from all surveyed patients and detailed inferential analysis results for research questions *R1*-*R5* are provided in the S1 Data.

## 4. Discussion

We observed in this study that a significant proportion of patients (40.44%) preferred to make a first visit to specialized healthcare facilities (public or private). This finding is consistent with the work by Narang [29], Das et al. [30], Kelen et al. [31], and Rao & Sheffel [32], who found that a majority of patients bypassed primary and secondary care facilities to directly seek care from specialists at higher level facilities. This practice adversely affects the hierarchy in patient flow across the public healthcare facility network and can lead to both overcrowding at facilities offering a higher level of care and underutilization of lower-level facilities. Therefore, our findings for an urban Indian district, in conjunction with the findings by Rao & Sheffel [32] for primary healthcare centres (PHCs) in a rural Indian region, emphasize the need for a comprehensive pan-Indian investigation into the reasons for bypassing public primary and secondary healthcare facilities. Rao & Sheffel [32] discussed that improving structural quality of PHCs alone is unlikely to suffice, and that improvement in provider attitudes and the quality and quantity of time spent with patients is likely to be required. Our findings also support this in two ways: first, from the HSB survey, we find that patients choose to visit facilities where they have had prior good experiences not only in terms of adequate infrastructure, but also in terms of quality of care, and provider attitudes (reflected in, for instance, longer consultation durations). Secondly, observations from the surveyed facilities indicate the low operational utilization of the doctors, given their low consultation times. Barik & Thorat [8] also commented that quality health services in terms of both infrastructure and patient-provider interactions, either provided via public or appropriately regulated private channels, can help achieve universal access to healthcare for everyone.

From the perspective of gender, female patients indicated a higher preference for private healthcare providers. This is consistent with findings from Kenya, where Keesara et al. [33] also reported that women avoided public facilities owing to long waiting times and impolite care providers. They also were willing to pay more for private care. Grosse Frie et al. [34] collected information on pathways of women seeking diagnostic services for breast-related symptoms and concluded that women visited private clinics due to shorter times to the first consultation. Patel & Chauhan [11] emphasized introducing more women-friendly measures to tackle gender-related discrimination in health care utilization at public healthcare facilities.

We also observed that a significant proportion of patients (80.14%) preferred visiting private healthcare facilities which lead to their incurring significantly higher medical expenses over visiting public facilities for specialized medical services required to treat serious and/or chronic illnesses. Rout et al. [9] provided quantitative evidence on the utilization of public and private health facilities, and its variations across states of India. This study supported our findings in showing that the public sector is not the preferred choice for the majority of the population across Indian states. Similarly, patients with higher incomes were 4.34 times more likely to visit private healthcare facilities in comparison to patients with lower incomes. Given India’s large population, it may not be feasible for public healthcare facilities alone to cater to the healthcare needs of the entire populace. However, our study illustrates that an investigation into factors that discourage patients (in particular, those with low annual incomes) from seeking care at public healthcare facilities, especially for illnesses of perceived high severity, is warranted. This can help improve the utilization of existing public facilities and the financial burden on low income patients.

The perception of getting better quality of care at a subset of the network of public facilities in a region contributes to significant variations in the utilization of public medical resources at a similar level of care. Dissatisfaction with the healthcare facility was one of the major reasons also expressed in Charles et al. [35]. Further, patients with higher education levels were less likely to visit a public healthcare facility (0.38 times less likely) in comparison to patients with no formal education. Similar observations were reported in other lower and middle income countries [36–38]. We also observed that patients with undergraduate and/or post-graduate degrees were 0.18 times and 0.21 times less likely to visit a traditional healer in comparison to patients without any formal education. Hahn & Truman [39] and Zajacova & Lawrence [40] also discussed how higher levels of education raised awareness among patients and increased the likelihood of seeking formal care in other countries.

### 4.1 Public health policy insights

Public health planning authorities can use the findings of our study to formulate a policy framework designed to improve the utilization of public healthcare services. Preferences for the private health sector and non-utilisation of public primary care are signals that the public primary health system is failing to meet the health needs of the population. This can include, for example, a campaign to increase awareness among the general public regarding the role of the public primary healthcare system in urban areas, including dispensaries, PUHCs and polyclinics. Targeted campaigns may also be required. For example, our study indicates that patients with lesser education and without any source of income preferred visiting traditional healers or took medication without consulting a formal care provider. Targeted programs can help improve awareness among these groups regarding the provision of free or nominally priced care at Indian public facilities.

In addition to such campaigns, a concerted effort to improve overall quality of care will be required, in terms of infrastructure, availability of drugs and medical equipment at these facilities. Perhaps more importantly, sensitizing providers to improve their patient care attitudes (such as building and improving trust among female patients), minimizing absenteeism, and increasing patient consultation times to reasonable levels, as explored in Shoaib & Ramamohan [27], are likely to be required.

Improved quality of care at primary and secondary care facilities may need to be accompanied by the implementation and enforcement of an effective referral mechanism across the public healthcare network in Southwest Delhi to alleviate the problem of overcrowding at higher levels of care. Similar mechanisms exist in developed nations such as Britain, France, Germany, Singapore, and South Korea where deviating from predefined referral pathways may lead to penalties for non-urgent cases in terms of delayed reimbursement, higher copayment, or longer wait times [41, 42].

### 4.2 Strengths and limitations

We collected patient HSB data from respondents surveyed at multiple primary and secondary public healthcare facilities based in a dense urban metropolitan city with a significant number of private and public healthcare facilities to determine significant factors influencing patients to visit public healthcare facilities. Further, our study provides a comprehensive quantitative analysis of the HSB of these patients regardless of their presenting condition while satisfying nearly all the methodological criteria detailed in S1 Table. Our study also elicited information regarding patient pathways and determinants of utilization of the surveyed facilities, which may also be indicative for other urban Indian facilities. These include pathways other than those leading to the surveyed facilities adopted by patients.

A key limitation of this work is that the HSB analyses are based on the survey conducted in the South-west Delhi district alone. Thus, the findings may not be representative of the entire population of New Delhi or other urban regions. We only interviewed patients who received care at the surveyed facilities, without surveying patients at other facilities (e.g., privately run healthcare facilities or tertiary care hospitals) or households. Thus, conducting the above types of studies in other regions of India forms a key avenue of future research. Our study provides a template for conducting such studies, analyses that can be done from the data collected via these studies, and the insights that can be generated–not only for India, but also for other developing nations with a similarly complex healthcare landscape.

## 5. Conclusion

This article examines, via a cross-sectional survey, the HSB of patients visiting primary and secondary public healthcare facilities in an urban Indian district. Based on our survey responses, logistic regression was used to model the association between various aspects of patient HSB and their socioeconomic and demographic attributes. The analyses conducted in the study provided quantitative evidence for the association of HSB with socioeconomic and demographic factors such as annual income, education level, occupation, gender, age and other factors such as perception of expected wait time and cleanliness at healthcare facilities, expected quality of care, and behavior of service providers. The survey has yielded information for public health policymakers regarding specific issues associated with these health systems that require redressal so that patients’ perception of quality of care at these facilities and consequently their utilization can be improved.

## Supporting information

S1 Table Summary of previous literature. (XLSX)

S1 Fig Survey questionnaire for recording HSB of participants. (TIF)

S1 Text Survey questionnaire design and description. (DOC)

S2 Text Survey sample size estimation. (DOC)

S3 Text Statistical operations around study research questions. (DOC)

S1 Data Survey responses and statistical model parameter estimates. (XLSX)
