# Supplementary figures and images for: Healthcare seeking behavior among patients visiting public primary and secondary healthcare facilities in an urban Indian district: A cross-sectional quantitative analysis
Source: PLOS Glob Public Health. 2023 Sep 5;3(9):e0001101. doi: 10.1371/journal.pgph.0001101 (PMC10479939; doi:10.1371/journal.pgph.0001101)

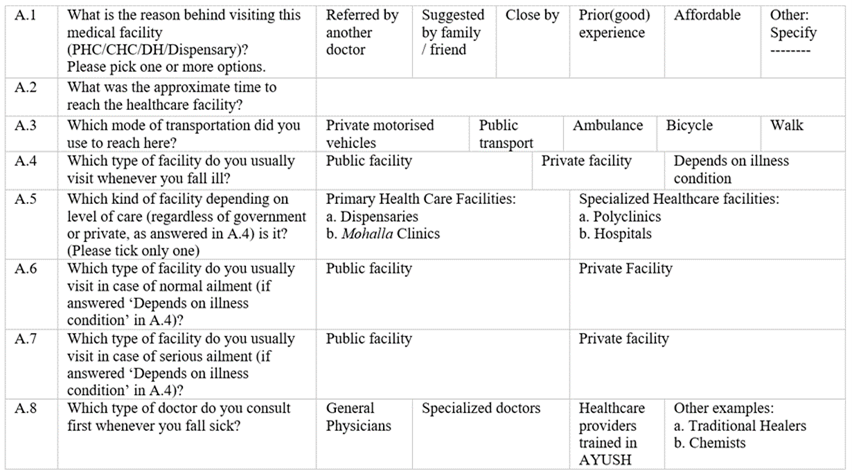

Supplement: S1 Fig — (TIF) [file pgph.0001101.s002.tif]
