# Supplementary material for: Healthcare seeking behavior among patients visiting public primary and secondary healthcare facilities in an urban Indian district: A cross-sectional quantitative analysis
Source: PLOS Glob Public Health. 2023 Sep 5;3(9):e0001101. doi: 10.1371/journal.pgph.0001101 (PMC10479939; doi:10.1371/journal.pgph.0001101)
Supplement: S1 Text — (DOC) [file pgph.0001101.s003.doc]

# Survey questionnaire design and description

We designed survey questionnaires for patients in the English and Hindi languages. We divided the survey questionnaire into 2 sections with 15 questions enquiring about: (a) HSB (including reasons leading to healthcare seeking, types of healthcare services utilized, and average time to reach healthcare facility, among others - 8 questions), and (b) demographic details (7 questions). Demographic details collected included patient gender, age, marital status, annual income, number of children, employment status, and education level. We present the first part of the survey questionnaire in Fig S2 (additional file).

We briefly discuss questions presented in Fig S2 meant to examine HSB of patients. First, we explored motivating factors for seeking care from primary and secondary public healthcare facilities in a large metropolitan city such as New Delhi, wherein a large number of private alternatives are available.

*Fig S2:* Survey questionnaire for recording HSB of participants.

Note: AYUSH = Ayurveda, Yoga and Naturopathy, Unani, Siddha, Homeopathy.

First, we explored motivating factors for seeking care from primary and secondary public healthcare facilities – dispensary, PUHC, or polyclinics in a large metropolitan city like Delhi with availability of significantly large number of private alternatives. We allowed participants to choose more than one option in their response to this question (question A.1). Next, we recorded the time it took patients to reach the healthcare facility using different modes of transportation (questions A.2 and A.3) in order to understand how accessible these facilities are to the general population.

Subsequently, via question A.4, we attempted to determine the type of healthcare facility patients – public or private - preferred to visit first upon falling ill. We note here that while public healthcare facilities offer free medical services for every resident per the guidelines of the Government of Delhi, private healthcare facilities operate autonomously and provide medical care on a chargeable basis with significant variance in both quality of care provided, and the fees charged. For patients who indicated that their preference for visiting a particular type of healthcare facility was dependent on their illness condition, we recorded their responses separately via questions A.6 and A.7 depending upon their perception of the severity of their illness.

We next examined, via question A.5, what level of healthcare facility – primary care or more specialized facility, regardless of whether they are public or private facilities - patients preferred to visit first upon falling ill. Under primary care facilities, we included ‘*mohalla’* (neighbourhood in Hindi) clinics or dispensaries, and we included polyclinics and hospitals under specialized facilities. This question was meant to quantitatively determine the likelihood of patients in an urban metropolitan city such as New Delhi to bypass lower-level facilities for seeking care directly at specialized facilities. Finally, via question A.8, we attempted to determine the preference of patients for a specific type of doctor to visit upon first falling ill (general physicians, specialized doctors, doctors formally trained in traditional forms of medicine, and others including informal traditional healers and chemists), regardless of which type of facility the provider is situated in (public/private, primary/higher level of care).
