# Supplementary material for: Healthcare seeking behavior among patients visiting public primary and secondary healthcare facilities in an urban Indian district: A cross-sectional quantitative analysis
Source: PLOS Glob Public Health. 2023 Sep 5;3(9):e0001101. doi: 10.1371/journal.pgph.0001101 (PMC10479939; doi:10.1371/journal.pgph.0001101)
Supplement: S2 Text — (DOC) [file pgph.0001101.s004.doc]

# Survey sample size estimation

We estimated the sample size for the survey based on pilot survey responses from forty participants. For each question *N_1_, N_2_, N_3, …_. , N_s_* included in the questionnaire, we first estimated its corresponding sample size depending upon whether the response variable for the question was a discrete or continuous variable.

For questions with continuous response variables, the sample size *S_c_* was estimated using equation 1 below.


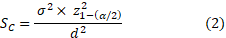


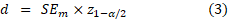


Here,
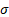
 is the standard deviation estimated for participant responses during the pilot survey for each question with a continuous response variable,
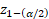
 is the standard normal random variable quantile associated with a level of significance
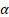
,
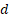
 is the margin of error and
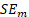
is the standard error of the mean.

For questions with discrete response variables, the sample size
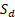
 was estimated using equation 3 below.


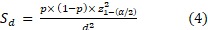


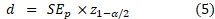


Here,
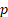
 is the proportion estimated for each category of the discrete response variable during the pilot survey, and
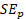
is the standard error of the proportion estimate
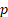
.

After calculating
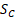
 and
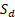
, we estimated additional factors such as the population size effect, sampling effect, response rate, and eligibility rate using expressions provided below and incorporated all these factors in estimating the final sample size for the survey.

Population size effect (
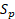
)
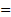

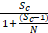
 (
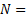
 study area population)

Sampling effect (
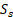
)
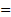

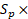
 design factor

Response rate (
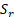
)
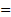

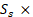
expected response rate in pilot survey

Eligibility rate (
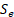
)
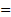

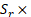
 expected proportion eligible

Final sample size
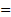

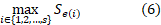


We repeated the above calculations for each question included in the survey questionnaire and then estimated final sample size using equation 5. The final sample size for the patient survey was found to be four hundred and forty-nine.
