# Supplementary material for: Healthcare seeking behavior among patients visiting public primary and secondary healthcare facilities in an urban Indian district: A cross-sectional quantitative analysis
Source: PLOS Glob Public Health. 2023 Sep 5;3(9):e0001101. doi: 10.1371/journal.pgph.0001101 (PMC10479939; doi:10.1371/journal.pgph.0001101)
Supplement: S3 Text — (DOC) [file pgph.0001101.s005.doc]

# Statistical operations around study research questions

We formulated five research hypotheses including four BLR-based hypotheses.

*Research question (R1).* What socioeconomic and demographic factors are associated with a patient visiting a public or a private healthcare facility?

The null and alternate hypotheses associated with the above research question are as follows.


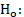
Socioeconomic and demographic variables do not influence a patient’s choice of visiting a public or a private healthcare facility.


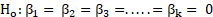


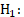
Socioeconomic and demographic variables influence a patient’s choice of visiting a public or a private healthcare facility.


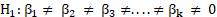


Similar to *R1*, we formulated *R2*, *R3*, and *R4*. We provide the null and alternate hypotheses for *R2*, *R3* and *R4* after we list *R2*, *R3*, and *R4* below.

- *R2.* What socioeconomic and demographic factors are associated with patients visiting a public or a private healthcare facility to seek care for illness conditions of perceived mild severity?
- *R3*. What socioeconomic and demographic factors are associated with patients visiting a public or a private healthcare facility in the case of illness conditions of perceived high severity?
- *R4.* What socioeconomic and demographic factors are associated with a patient visiting a primary or a specialized healthcare facility for their first visit upon falling ill?

Now to answer *R1*, we first selected the reference categories for the predictor and response variables. The response variable had two categories: (a) public healthcare facility (labelled 1), and (b) private healthcare facility (labelled 0). We chose ‘private healthcare facility’ as the reference category, with the dependent variable being whether a person visits a public or a private healthcare facility given the set of attributes in Table 3. We treated all predictor variables as discrete, including patient age and annual income. We observed that five out of seven variables, i.e., (a) gender, (b) education level, (c) employment status, (d) annual income, and (e) number of children were significantly influencing patient choices. We present parameter estimates of significant independent variables in Table 4.

Thus, the BLR model with significant socioeconomic and demographic predictor variables is given in equation (7) below.


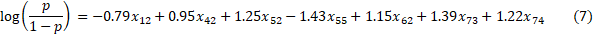


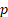

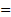
 probability of visiting a public healthcare facility


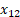

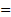
 1, if the respondent is female


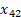

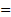
 1, if the respondent has completed 10^th^ grade


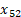

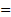
 1, if the respondent has annual income between USD 11,500 – USD 23,000


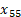

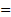
 1, if the respondent has annual income above USD 46,000


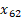

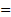
 1, if the respondent is homemaker


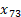

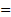
 1, if the respondent has 2 children


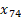

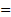
 1, if the respondent has 3 or more than 3 children

*Research question (R2).* The null and alternate hypotheses associated with *R2* are given below.


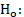
Socioeconomic and demographic attributes do not influence a patient’s choice of visiting a public or a private healthcare facility in seeking care for illness conditions of perceived mild severity.


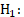
Socioeconomic and demographic attributes influence a patient’s choice of visiting a public or a private healthcare facility in seeking care for illness conditions of perceived mild severity.

The BLR model for *R2*, with significant variables alone, is given below.


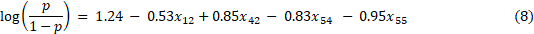


p
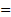
 probability of choosing a public healthcare facility


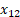

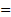
 1, if the respondent is female


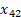

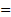
 1, if the respondent has completed 10^th^ grade


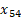

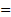
 1, if the respondent has annual income between USD 23,000 – USD 46,000


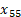

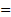
 1, if the respondent has annual income above USD 46,000

*Research question (R3).* The null and alternate hypotheses associated with *R3* are given below.


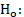
Socioeconomic and demographic attributes do not influence a patient’s choice of visiting a public or a private healthcare facility in seeking care for illness conditions of perceived high severity.


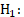
Socioeconomic and demographic attributes influence a patient’s choice of visiting a public or a private healthcare facility in seeking care for illness conditions of perceived high severity.


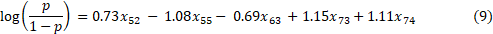


p
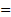
 probability of choosing a public healthcare facility


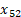

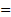
 1, if the respondent has annual income is between USD 11,500 – USD 23,000


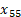

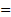
 1, if the respondent has annual income greater than USD 46,000


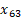

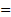
 1, if the respondent is employed


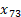

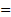
 1, if the respondent has 2 children


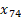

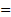
 1, if the respondent has more than 2 children

*Research question (R4).* For R4, of the two complementary categories for this response variable - (a) primary healthcare facilities, and (b) specialized healthcare facilities - we chose the latter as the reference category. The null and alternate research hypotheses are given below.


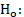
Socioeconomic and demographic attributes do not influence a patient’s choice of visiting a primary or a specialized healthcare facility for their first visit.


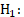
Socioeconomic and demographic attributes influence a patient’s choice of visiting primary or a specialized healthcare facility for their first visit.

The BLR model for *R4*, with significant variables alone, is expressed as:


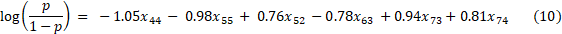


p
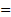
 probability of choosing a primary level healthcare facility


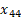

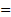
 1, if the respondent has undergraduate degree


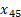

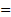
 1, if the respondent has post-graduate degree


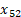

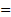
 1, if the respondent has annual income between USD 11,500 – USD 23,000


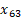

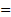
 1, if the respondent is employed


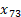

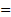
 1, if the respondent has 2 children


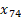

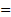
 1, if the respondent has more than 2 children

We now discuss the inferential analysis around research question *R5*, which involves multinomial regression modeling.

*Research question (R5).* The null and alternate hypotheses for *R5* are given below.


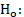
Socioeconomic and demographic attributes of patients are not associated with their choice of medical practitioner type.


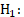
Socioeconomic and demographic attributes of patients are associated with their choice of medical practitioner type.

The predictor variables are described in Figure 2, and we chose ‘general physician’ as the reference category and built three individual BLR models with respect to the reference category. Six independent variables - marital status, age, education level, annual income level, number of children, and employment status - turned out to be significant predictors. We report the parameters of significant independent variables for all three models in separate rows in Table 5.

The MLR models, with significant variables alone, are provided below.


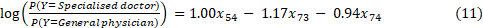


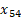

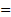
 1, if the respondent’s annual income is between USD 23,000 – USD 46,000


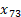

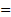
 1, if the respondent has two children


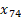

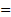
 1, if the respondent has three or more than three children


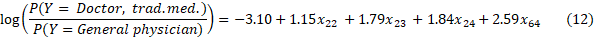


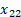

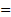
 1, if the respondent is between 30-39 years of age


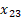

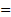
 1, if the respondent is between 40-49 years of age


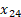

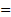
 1, if the respondent is between 50-59 years of age


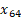

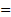
 1, if the respondent’s is unemployed


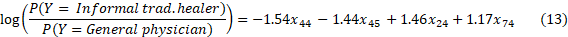


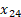

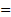
 1, if the respondent is between 50-59 years of age


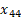

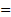
 1 if the respondent has undergraduate degree


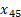

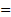
 1 if the respondent has postgraduate degree


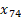

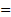
 1 if the respondent is unemployed
